# 

**Published:** 1886-12-11

**Authors:** 


### The Hospital.

**An Institution, Family, and Parochial Journal of Hospitals, Asylums, and all Agencies for the Care of the Sick, Criticism and News.**

SATURDAY, DECEMBER 11TH, 1886.

WE have received the following books for review:—

CASSELL & CO.
The Eye, Ear, and Throat. By Henry Power, F.R.C.S., George P. Field, and John S. Bristowe, M.D., F.R.S.
The Skin and Hair. By Malcolm Morris, F.R C.S.
The Influence of Clothing on Health. By Frederick Treves, F.R.C.S.

R. L. HOMES, Glasgow.
The Abuse of our Medical Charities. By James Erskine, M.A., M.B.

KEGAN PAUL & CO.
Pulmonary Phthisis. By S. Jacquod. Translated by Montague Lubbock, M.D.
Bacillary Phthisis. By Germain Sée.
Microbes, Ferments, and Moulds. By E. L. Trouessart.
Lectures on Nursing. By Eva Luckës.

E. & S. LIVINGSTONE, Edinburgh.
The Monthly Nurse. By H. Aubrey Husband, M.B.

MACMILLAN & CO.
Disorders of Digestion. By T. Lauder Brunton, M.D., F.R.S.
Diseases of Tropical Climates. By William Campbell Maclean, M.D., C.B.
Hygiene of the Vocal Organs. By Morell Mackenzie, M.D.

SMITH, ELDER & CO.
School Hygiene. By Robert Farquharson, M.P.
What Shall be my Practice? By E. Diver, M.D.
First Aid to the Injured. Translated from the German by H.R.H. Princess Christian.
Lectures on Dietetics. By Sir Wm. Roberts, M.D., F.R.S.
Clinical Manual. Edited by James Finlayson, M.D.

### THE QUILTER PRIZES.

MR. W. CUTHBERT QUILTER, M.P., has offered £50 in prizes, with the view of encouraging all engaged in hospital and asylum work to promote efficiency and economy in the management of these institutions. The object of these Prizes, and a full description, will be found set forth on page 115 of THE HOSPITAL of the 13th ult., with the sections and subjects suggested. The following are the conditions and the subject of the first competition:—

THE CONDITIONS.

1. We shall offer two Prizes of varying amounts, according to the importance attached to the subject of the competition. A cheque will be sent on the day the paper is published in which the results of each competition are given.

2. In all cases the names and addresses of the competitors must be forwarded, even when a *nom de plume* is used.

3. The answers must be addressed to "The Prizes Editor, THE HOSPITAL, Norfolk House, Norfolk Street, Strand," not later than the 15th December next, at noon. All communications must be written on one side of the paper only.

1. A PRELIMINARY COMPETITION.

At the outset we ask for suggestions from each of the classes of readers mentioned on page 115, as to the subjects in each section which they themselves consider to be the best to select for the purposes of this competition. The suggestion, under any of the heads specified, which seems to us the most practical and in all respects the best will receive the first prize of £3, and the next best suggestion the second prize of £2.

### THE CHILDREN'S CORNER.

### OUR PASTIMES.

**Children's Quarterly Prize Competition.**

Began .. .. .. .. .. 2nd Oct., 1886

Ends .. .. .. .. .. 25th Dec., 1886

PRIZES.

1st .. .. .. .. Thirty Shillings

2nd .. .. .. .. Twenty „

3rd .. .. .. .. Ten „

4th .. .. .. .. Five „

will be awarded to the four competitors who shall have sent in, during the thirteen weeks, the largest number of correct answers to our pastimes.

**Eleventh Competition.**

No. 55. I have a piece of paper of the following shape:

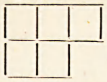

containing, as you see, five little squares. I want you to tell me how, by making *two* cuts, I can make the three pieces form an exact square. There must be no odd piece over. (Answers should enclose a piece of paper with the necessary cuts.)

No. 56. Evolve, if you can, a little French sentence out of the following:

o o o o o

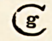

No. 57. Can you tell me which train from Victoria is the most difficult for an intending passenger to catch?

No. 58. Diamond Puzzle. 1, a consonant; 2, a negative; 3, an adhesive; 4, portions which tell you what the whole is like; 5, the synonym for an institution; 6, a dead language; 7, a word meaning distant; 8, a consonant. The centre letters read downwards, and the word in the fifth line constitute the title of a most useful society.

No. 59. Double Acrostic. The Portuguese word for "lord"; a river of Russia; a domestic animal; something like pitch; an interjection; a word which means to hasten. The initials and the finals form the titles of two persons most indispensable in every hospital.

No. 60. Why was it impossible for the Children of Israel to have starved in the desert?

**Results of Ninth Competition.**

No. 44. The answer here is "a book." Nearly all my little contributors guessed this. How is it, Tina, you did not?

No. 45. Filomel says that my French puzzles are too difficult for her. Well, I must confess that this was not altogether easy, though Alsie, T.K., and Tina come out victorious. The puzzle is to be thus "construed." G, in French, is approximately pronounced *Zhay*, which gives us *J'ai*. The G is crossed by I or G (*Zhay*) *traversé par I* (par *ee*=Paris). The "sans," coming under (*sous*) "liers," gives us "sans souliers"; so the whole reads, "J'ai traversé Paris sans souliers." Strictly speaking, the "sans," should be "tied" (*lier*, to bind) within the tail of a written capital G, but this was not possible in print, and as I set it, it was less difficult for my little guessers to unriddle. A friend tells me that he believes that the enigma actually formed part of a letter sent in cipher from the Duchess of Montpensier to her husband in perilous times.

No 46. This reads as follows:

If the grate be ("B") empty (m t)
Put coal on (:),
If the grate be ("B") full,
Stop (.) putting coal on (:).

The puzzle is a very old one.

No. 47. Beginning with the bottom line, and reading backwards, we get the well-known nursery rhyme, "Ride a cock horse to Banbury Cross, etc." It was very generally guessed.

No. 48. I was both pleased and surprised to see everyone right here. The thief, after abstracting the money, arranged the remainder thus:

| | | |
|---|---|---|
| 3 | 9 | 3 |
| 9 | | 9 |
| 3 | 9 | 3 |

| | | |
|---|---|---|
| 5 | 5 | 5 |
| 5 | | 5 |
| 5 | 5 | 5 |

Mikado, T.K., Alsie, and True Blue give other orders which would have afforded the same deception.

The competition is proving a very close one, and promises, to use a sporting term, to have an exciting finish. The puzzles and conundrums which I have set have been by no means of the easiest kind, and the general accuracy of the answers therefore speaks well for the ingenuity and thought which my little friends have exercised upon them. Will Florrie please to address her envelope to the "Puzzle Editor"? Her omission to do this has caused the error to which she draws my attention.

All right—Alsie.
Four right—T. K., Tina, Retsibsi, Mikado, Florrie.
Three right—Jimmy, True Blue, Filomel, Little Mary.
Two right—Bismarck, Nonsuch.

Florrie has been duly credited with the four former ones.

Answers, having the actual name and address (to which a pseudonym may be added for publication), must have "The Children's" Coupon attached (see advertisement pages), and be addressed to the "PUZZLE EDITOR," THE HOSPITAL, Norfolk House Norfolk Street, Strand, W.C., not later than Thursday, 16th December. Results will appear in our issue of the 25th December.

### "THE HOSPITAL" CHARITY BOX.

OUR "Charity Box" is intended to afford a little practical help to the hospitals. Each week we propose a word for "anatomical dissection," and the first 150 competitors who extract the largest number of words from it receive the following prizes:—

1st .. .. .. One Guinea.
2nd .. .. .. Half a Guinea.
3rd .. .. .. Five Shillings.

The above Prizes will be repeated for every additional 150 competitors who may enter each week

ELEVENTH COMPETITION.

Compose the largest number of words you can from "PHYSICIAN."

*Observe:* Proper names, abbreviations, foreign, Latin, and obsolete words are barred.

Repetitions of similarly spelt words and of letters (unless they repeat in the word) are disallowed.

Plurals allowed, but not the possessive in 's or s'.

Prefixes and affixes do not count independently of words.

Write only on one side of the paper, and total your words.

Append your real name and address, adding whether Mr., Mrs., or Miss, etc.

All replies, addressed "Charity Box," THE HOSPITAL, Norfolk House, Norfolk Street, W.C., must arrive by Friday, Dec. 17th. Results will appear Saturday, Dec. 25th.

*Every Competitor is required to cut out and enclose with his list the "Charity Box" Coupon* (see advertisement pages), *and a shilling entrance fee* (postal orders only, *crossed Lloyd's, etc., Banking Company, Limited*, THE HOSPITAL *Account.*)

The proceeds resulting from each "Charity Box" competition will be funded until the end of December 1886. The total sum available for distribution, and the names of the hospitals selected for donations, will appear on Saturday, January 8, 1887. The distribution of the proceeds will be at the Editor's sole discretion.

RESULTS OF NINTH COMPETITION

The "Doctor" Competition has resulted as follows:—

| | Accepted Score. |
|---|---|
| Miss G. A. Dundas, 2, Upper Brook Street, W. | 31—1st Prize. |
| Mr. Wallace A. Smith, 7, Crane Street, Chester .. .. .. .. .. .. .. .. | 33—2nd Prize. |
| Miss Walmisley, 7, Scarsdale Terrace, Kensington .. .. .. .. .. .. .. | 29 -3rd Prize. a "tie." |
| Mrs Shapley, Bryngaru, Hatherley Road, Sidcup .. .. .. .. .. .. .. | 29 -3rd Prize. a "tie." |

The prizes have been forwarded to the winners.

Mr. Wallace A. Smith having previously taken the first prize in the Abernethy Competition, is placed second in accordance with our rule. We redeem the promise we made last week by publishing the following list of words made by Mr W. A. Smith from the word "Doctor."

*Cod, coo, coo'd, coot, cor, cord, cot, crot, do, doo, door, dor, dot, O, oc, od, or, orc, ord, odor, ort, roc, rod, rood, root, rot, to, tod, to-do, too, tor, tore, trod.*

We have barred "co," which is either a prefix or an abbreviation. Such words as roco, roto, oct, octo are disallowed by our rules. We have admitted the American spelling "odor" for the reason given in THE HOSPITAL of the 20th ult.

**Special Notice.**

In the interest of non-successful competitors, we have decided not to award the First Prize more than once to a winner in any one quarter. In the event of a competitor again making the highest accepted score, he will be placed in the second place, and, if a third time highest, in the third place.

**Notices to Competitors.**

A Lancashire Lass.—Names of persons, counties, countries, towns, mountains, and rivers would certainly be proper names, and as such, they are barred. If by names of "animals and flowers," you mean such words as tiger, zebra, lily, anemone, we should of course allow them.

Miss Walmisley.—The letters of the alphabet are not counted as "words."

Miss A. Clift.—It is only the first prize that we bar being won twice by the same competitor in any one quarter. You are entitled to win the second and third any number of times.

Mr. D. Cherrett.—We thank you for your very courteous letter. Several correspondents have written us expressing their satisfaction in our judgments, both winners and non-winners.—Only dictionary words are allowed.

### THE IN- AND OUT-PATIENTS' HOSPITAL DIARY.

This Diary is so arranged as to make some portion of it appear every week, and the whole in each monthly part. It has been very difficult to compile, and corrections will at all times be gratefully received. It has been suggested that similar information about the larger Provincial and Scotch Hospitals would be useful to many readers. We should be glad to hear if this is felt to be the case.

### GENERAL HOSPITALS.—*continued.*

| Hospitals and Terms of Admission. | Physicians, Surgeons, and Medical Officers, with Days and Hours of Attendance. |
|---|---|
| St. Mary's Hospital, Cambridge Place, Paddington, W. Sec., Mr. Pietro Michelli<br>In-patients are admitted by letter of recommendation. Urgent cases and accidents free at all times. Out-patients require a letter. Electrical treatment Tu. and F. 2 | *In-Physicians*, Sir Edward Sieveking, Tu. F. 1.45; Drs. Broadbent, M. Th. 1.45; Cheadle, W. 1.45, S. 9.30<br>*In-Surgeons*, Messrs. Norton, M. Th. 1.45; Owen, Tu. F. 1.45; H. Page, W. S. 1.45<br>*Out-Physicians*, Drs. D. B. Lees, W. S.; S. Phillips, M. Th; R. Macguire, Tu. F. Hour, 1.30<br>*Out-Surgeons*, Messrs. W. Pye, M Th.; A. J. Pepper. Tu. F.; A. Q. Silcock, W. S. Hour, 1.30 |
| St. Thomas's Hospital, Westminster Bridge Road, S.E. Steward, Mr. F. Walker<br>Accidents and emergencies free at all times Other cases by Governor's letter, but, where unobtainable, application should be made to the Steward by letter, or personally between 10 and 4. Vaccination on W. at 11.30 | *In-Physicians*, Drs. Bristowe, Tu. F.; Stone, M. Th.; Ord, M. Th.; J. Harley, Tu. F.; Gervis, Tu. F. Hour, 2<br>*In-Surgeons*. Messrs. Sydney Jones, Tu. F.; Croft, M. Th.; Sir W. MacCormac, M. Th. Hour, 2<br>*Out-Physicians*, Drs. Payne, Tu. F; Sharkey, M. Th.; Gulliver, W. S. Hour, 1.30<br>*Out-Surgeons*, Messrs. Clutton, Tu. F.; Anderson, W. S.; Pitts, M. Tu. Hour, 1.30 |
| Tottenham Training Hospital, Tottenham, N. Director, Dr. Laseron.<br>Free. Accidents and emergencies admitted at all times | *In-Physician*. Dr. Rasch, Tu. F. 3 to 5<br>*In-Surgeons*, Drs. Lichtenberg, M. Th. 3 to 5; E. H. May, M. Th. 3 to 5<br>*Out-Surgeon*, Dr. Lloyd Smith, M. W. 11 to 2; *Men* only, W. 7 to 8 p.m. |
| University College Hospital (or North London), Gower Street, W.C. Sec., Mr. N. H. Nixon.<br>Accidents and emergencies free. All other cases by Governor's letter of recommendation | *In-Physicians*, Drs. W. Fox, Tu. Th. 2, S. 9.30; Ringer, M. W. F. 1.30; Bastian, M. 2.30, W. 10, F. 2.30; F. Roberts, Tu. 1.15, Th. 1.30, S. 9.30<br>*In-Surgeons*, Messrs. B. Hill, Tu. 2.15, W. 2, F. 2.15; C. Heath, M. W. F. 2; M. Beck, Tu. Th. S. 2<br>*Out-Physicians*, Drs. Gowers, W. S.; Poore, Tu. F.; Barlow, M. Th. Hour, 1.30<br>*Out-Surgeons*, Messrs. Barker, M. Th.; Godlee, Tu. F.; Horsley, W. S. Hour, 1.30 |
| West London Hospital, Hammersmith Road, W. Sec. and Supt., Mr. R. J. Gilbert.<br>Accidents and emergencies free at all times. Other cases require a letter of recommendation. New cases must attend at 1.30 | *In-Physicians*, Drs. G. Rogers, D. W. Hood, F. Drewitt<br>*In-Surgeons*, Messrs. C. Keetley, F. Edwards, W. B. Clarke<br>*Out-Physicians*, Drs. Herringham, M. Th.; Ball, Tu. F.; S. Taylor, W. S. Hour, 2<br>*Out-Surgeons*, Messrs. Ballance, Tu. F.; Weiss, M. Th.; Wainewright, W. S. Hour, 2 |
| Westminster Hospital, Broad Sanctuary, S.W. Sec., Mr. S. M. Quennell.<br>Accidents and urgent cases free at all times. Other cases require a letter of recommendation | *In-Physicians*, Drs. Sturges, M. Th. S.; Allchin, M. Tu. F.; Donkin, Tu. W. S. Hour, 1.30<br>*In-Surgeons*, Messrs. Cowell, M. Tu. Th.; Davy, M. Tu. F.; Macnamara, M. W. S. Hour, 1.30<br>*Out-Physicians*, Drs. De H. Hall, M. Th.; A. H. Bennett, Tu. F.; Murrell, W. S. Hour, 1.30<br>*Out-Surgeons*, Messrs. Cooke, M. Th.; Bond, Tu. F.; Barrow, W. S. Hour, 1.30 |

### II.—SPECIAL HOSPITALS.

### CANCER.

| | |
|---|---|
| Cancer Hospital, Brompton, S.W. Sec., Mr. W. H. Hughes.<br>Entirely free to both in- and out-patients | *In- and Out-Surgeons*, Drs. A. Marsden, W.; H. Snow, M.; F. A. Purcell, F.; Messrs. F. B. Jessett, W.; C. Stonham, Th.; C. Jennings, Tu.; W. H. Elam, S. Hour, 2 |
| London Hospital, Whitechapel Road, E. | Daily, 1.30. *See* General Hospitals |
| Middlesex Hospital, Berners Street, W. | *In- and Out-Surgeon*, Mr. Morris, Th. 1.30 |
| St. Saviour's Hospital, Osnaburg Street, N.W. Sec., Mr. E. Poitiers.<br>By Governors' letters | *In-and Out-Physician*, Dr. A. Kennedy, 10.30 and 4 daily |

### CHILDREN'S DISEASES.

| Hospitals and Terms of Admission. | Physicians, Surgeons, and Medical Officers, with Days and Hours of Attendance. |
|---|---|
| Alexandra Hospital for Children, 18, Queen Sq., W.C. Sec., Mrs. Marsh.<br>For hip disease | *Physician*, Dr. Steavenson.<br>*Surgeons*, Messrs. H. Marsh Tu. F.; A. Bowlby, M. Th. Hour 4.30<br>(No out-patients) |
| All Saints' Children's Hospital, 4, Margaret Street, W.<br>For chronic joint diseases. | *Surgeon*, Mr. N. Smith.<br>(No out-patients) |
| Belgrave Hosp. for Children, 77 & 79, Gloucester Street, Pimlico. Hon. Secs., Capt. Stopford, and Rev. J. Storrs<br>By letters; accidents free | *In- and Out-Physicians*, Drs. W. Hope and W. Ewart, M. Th. 9<br>*In- and Out-Surgeons*, Messrs. C. T. Dent and Margerison, Tu. F. 9 |
| Charing Cross Hospital, West Strand, W.C. | *Out-Physician*, Dr. Nursby, W. S. 1.30 |
| Cheyne Hospital for Sick and Incurable Children, 46, Cheyne Walk, Chelsea, S.W. Sec., Miss D. Evans | *Physician*, Dr. G. V. Poore<br>*Surgeons*, Messrs. T. P. Bartlett and J. Macready attend alternate days in afternoons. (No out-patients) |
| East London Hospital for Children and Dispensary for Women, Glamis Road, Shadwell, E. Sec., Mr. Ashton Warner<br>By Governor's letter. Urgent cases and accidents are admitted free | *In-Physicians*, Drs. Donkin, M. Th.; Eustace Smith, T. F.; Crocker, F.<br>*In-Surgeons*, Messrs. Calder, W. F.; Parker, W. F.<br>*Out-Physicians*, Drs. Crocker, M.; Williams, Tu. Th; Coutts, W. F. Hours, 1 and 3<br>*Out-Surgeon*, Mr. Dunn, Tu. F. 1, 3 |
| Evelina Hospital for Sick Children, Southwark Bridge Road, S.E. Sec., Mr. T. S. Chapman.<br>Free, but Governor's letter takes precedence. In-patients (boys) between 2 and 10, (girls) between 2 and 12 | *In-Physicians*, Drs. F. Taylor and J. Goodhart<br>*In-Surgeons*, Messrs. H. G. Howse and R. C. Lucas<br>*Out-Physicians*, Drs. N. Tirard, M. Th. 9; F. Willcocks, Tu. F. 9<br>*Out-Surgeons*, Messrs. C. J. Symonds, S. 9; G. H. Makins, W. 9 |
| German Hospital, Dalston Lane, Dalston, E. | *In-Physician*, Dr. Rasch, M. Th. 9 |
| Great Northern Central Hospital, Caledonian Rd. N. | *Physicians*, Drs. Murray and Barnes, W. 2.30 |
| Grosvenor Hospital for Women and Children, Vincent Sq., S.W. Hon. Secs., Mr. A. J. Ram and Miss Day<br>By letter or payment | *In- and Out-Physicians*, Drs. R. Gibbons and Steavenson, Tu. W. Th. S. 2<br>*In- and Out-Surgeons*, Messrs. Smythe and Parker, Tu. W. Th. S. 2 |
| Hospital for Sick Children, 48 and 49, Great Ormond St., W.C. Sec., Mr. S. Whitford.<br>By Governors' letters. If without letter, the case of the applicant is investigated. In-patients between 2 and 12; Out-patients under 12 | *In-Physicians*, Drs. W. Cheadle, Tu. F.; Sturges, W. S.; Barlow, M. Th. Hours, 9 to 11<br>*In-Surgeons*, Messrs. H. Marsh, W. S.; E. Owen, W. S. Hours, 9 to 11<br>*Out-Physicians*, Drs. R. J. Lee and Abercrombie, M. Th.; Lubbock and Money, Tu. F. Hours, 8.30 to 10<br>*Out-Surgeons*, Messrs. Morgan and Pitts, W. S. 8.30 to 10 |
| King's College Hospital. Portugal Street, W.C. | *In- and Out-Physician*, Dr. T. C. Hayes, W. F. 1 |
| North Eastern Hospital for Children, Hackney Road, E. Sec., Mr. A. Nixon<br>By free ticket or small payment. | *In- and Out-Physicians*, Drs. W. Cayley, F. Turner, C. Semple, and W. Pasteur, M. W. Th. S. 1.30<br>*In- and Out-Surgeons*, Messrs. Waren Tay and Pollard, M. 1.30, F. 9 |
| Paddington Green Children's Hosp., Paddington Green. Sec., Mr. W. H. Pearce.<br>Admission free; boys under 12; girls under 14 | *In- and Out-Physicians*, Drs. Ogilvie, Tu. F.; G. Laycock, M. Th.; S. Phillips, W. S. Hour, 9.15<br>*In- and Out-Surgeons*, Messrs. W. W. Cheyne, M., S. Boyd, F. Hour, 9.15 |
| Royal Hospital for Children and Women, Waterloo Bridge Road, S.E. Sec., Mr. R. G. Kestin.<br>Out-Patients pay 1*d*. on each attendance. Boys not admissible, as out-patients, over 12, or, as in-patients, over 8 | *In-Physicians*, Drs. Park, M. Th.; Gulliver, Tu. F.; Price, W. S. Hour, 2<br>*In-Surgeon*, Mr. W. H. A. Jacobson, M. Th. Hour 2<br>*Out-Physicians*, Drs. Park, M. Th.; Dakin, W. F.; Hadden, Tu. S. Hour, 2<br>*Out-Surgeon*, Mr. E. O. Day, W. 2 |
| St. Mary's Hospital, Cambridge Place, Paddington. | *Out-Physician*, Dr. M. Handfield Jones, M. Th. 1.30 |
| St. Thomas's Hospital, Westminster Bridge Road, S.E | *Out-Physician*, Dr. Cory, W. S. 1.30 |

### CHILDREN'S DISEASES.—*continued.*

| Hospitals and Terms of Admission. | Physicians, Surgeons, and Medical Officers, with Days and Hours of Attendance. |
|---|---|
| Samaritan Free Hospital for Women and Children, Lower Seymour Street, W.; Branch, 1, Dorset St., W. Sec., Mr. G. Scudamore. In-patients must be between 2 and 12. Out-patients Department (free) is at Edward's Mews, Duke Street, W. | *In-Physician*, Dr. W. H. Day. Daily, 2<br>*In-Surgeon*, Mr. W. A. Meredith. Daily, 2<br>*Out-Physicians*, Drs. M. Prickett and Amand Routh, W. S. 1.30<br>*Out-Surgeons*, Messrs. W. A. Meredith and J. D. Malcolm, M. Th.; A. H. G. Doran and W. S. A. Griffith, Tu. F., 1.30 |
| Victoria Hosp. for Children, Queen's Road, Chelsea, S.W. Sec., Capt. W. C. Blount, R.N. Age of boys, 2 to 12 years; age of girls, 2 to 16. Admission for in-patients by subscriber's letter. Accidents and urgent cases free at all times | *In-Physicians*, Drs. J. Evans, M. Th. 2; Ridge-Jones, Tu. F. 2<br>*In-Surgeons*, Messrs. Pick, Tu. F. 9.15; Clutton, M. Th. 4.30<br>*Out-Physicians*, Drs. A. Venn, W. S. 9.30; C. Fox, M. Th. 1.30; F. D. Drewitt, Tu. F. 9; J. Philpot, M. Th. 9<br>*Out-Surgeons*, Messrs. F. Churchill, Tu. F. 10; W. Pye, M. Th. 9.30 |
| West London Hospital, Hammersmith rd, W. | Daily, 2. *See* General Hospitals |

### CONSUMPTION AND DISEASES OF THE CHEST.

| | |
|---|---|
| City of London Hospital for Diseases of the Chest, Victoria Park, E. Sec., Mr. T. Storrar Smith. By subscriber's letter available for 6 weeks. | *In-Physicians*, Drs. J. Thorowgood, S.; E. Smith, M.; J. Fothergill, W.; S. West, F.; G. A. Heron, W.; V. D. Harris, Tu. Hour, 2<br>*Out-Physicians*, Drs. V. D. Harris, Tu.; J. A. Ormerod, Th.; E. C. Beale, Tu. F.; B. Fenwick, W. S.; A. Money, W. S.; L. E. Shaw, M. Th. Hour, 2 |
| Hospital for Consumption, Fulham road, Brompton, S.W. Sec., Mr. H. Dobbin. Admission by letter of recommendation, but, where unobtainable, application may be made to the Secretary at the Hospital. | *In-Physicians*, Drs. E. S. Thompson, M. Th.; C. T. Williams, M. Th.; R. D. Powell, Tu. F.; J. Tatham, W. S.; R. E. Thompson, W. S.; F. T. Roberts, Tu. F. Hours, 2 to 4<br>*In-Surgeon*, Mr. R. J. Godlee, F.<br>*Out-Physicians*, Drs. T. H. Green, J. M. Bruce, M. Th.; J. K. Fowler, W. S.; P. Kidd and C. Y. Biss, Tu. F.; T. D. Acland, W. S. Hour, 12 |
| North London Hospital for Consumption and Diseases of the Chest, Mount Vernon, Hampstead, N. Sec., Mr. L. F. Hill. Admission by subscriber's letter available for 6 weeks. | *In-Physicians*, Drs. A. Evershed, Tu.; B. O'Connor, F.; D. Pidcock, F. Hour, 10.30<br>*Out-Physicians*, Drs. B. O'Connor, D. Pidcock, J. E. Squire. Daily, 2 (attend at Out-patients' Branch, 216, Tottenham Court Road, W.) |
| Royal Hospital for Diseases of the Chest, 231, City Rd., E.C. Sec., Mr. C. L. Kemp. By Governor's letter, available for six weeks | *In-Physicians*, Drs. Hensley, W.; Gilbart-Smith, M.; Finlay, Th.<br>*In-Surgeon*, Mr. A. Pearce-Gould, M. Tu. W. Th. F. 2, S. 9<br>*Out-Physicians*, Drs. White, Tu. F.; Pringle, M. Th.; O. Browne, W. S.; A. Davies, M. F.; H. Habershon, W. S.; E. Stewart, Tu. Th. Hour, 1; S. 9 |
| Royal National Hosp. for Consumption and Diseases of the Chest, Ventnor. Offices, 34, Craven St., Strand, W.C. Sec., Mr. E. Morgan. Admits only incipient cases by Governor's letter. | *In-and Out-Physicians*, Drs. J. G. Sinclair Coghill, and R. Robertson attend daily (at Ventnor) on out-patients at 11; on in-patients at noon<br>*In- and Out-Surgeons*, Messrs. Whitehead, Williamson |

### EAR CASES.

| | |
|---|---|
| Central London Throat and Ear Hosp., Gray's Inn Rd., W.C. Sec., Mr. R. Kershaw. Free, but when able, a small payment is expected | *In- and Out-Surgeons*, Mr. Lennox Browne, M. Th. 2.30; Dr. Orwin, W. S. 2.30; Dr. Grant, Tu. 5, Th. 2.30; Messrs. P. S. Jakins, M. W. 2.30; F. 5; T. Carmalt Jones, M. Th. S. 2.30 |
| Charing Cross Hospital, West Strand, W.C. | *In- and Out-Surgeon*, Mr. Cantlie, F. 9 |
| Great Northern Central Hospital, Caledonian Rd, N. | *In- and Out-Surgeon*, Mr. A. E. Cumberbatch, W. 9 |
| Guy's Hospital, St. Thomas's Street, S.E. | *In- and Out-Surgeon*, Mr. Purves, Tu. F. 12.30 |
| Hospital for Diseases of the Throat, Golden Square, W. Sec., Mr. W. T. Sharp | *Physicians*, Drs. Wolfenden, Tu. F. 2.30; Macdonald, Tu. F. 6.30; Bond, W. S. 2.30<br>*Surgeon*, Mr. T. M. Hovell, M. Th. 2.30 |

### EAR CASES—*Continued.*

| Hospitals and Terms of Admission. | Physicians, Surgeons, and Medical Officers, with Days and Hours of Attendance. |
|---|---|
| King's College Hospital, Portugal Street, W.C. | *In- and Out- Surgeon*, Mr. Pritchard, Th. 2.30 |
| London Hospital, Whitechapel Road, E. | *In- and Out-Surgeons*, Dr. Woakes, Mr. T. M. Hovell, S. 9 |
| Middlesex Hospital, Berners Street, W. | *In- and Out-Surgeon*, Mr. Hensman, Tu. 9 |
| National Hosp. for the Paralysed, etc., Queen Sq., W.C. | *In- and Out-Surgeon*, Mr. A. E. Cumberbatch, W. 2 |
| North West London Hospital, 18, Kentish Town Rd. | *Out-Surgeon*, Mr. Collier, M. 1.30 |
| St. Bartholomew's Hospital, Smithfield, E.C. | *Surgeon*, Mr. Cumberbatch, Tu. F. 2 |
| St. George's Hospital, Hyde Park Corner, S.W. | *In- and Out-Surgeon*, Sir W. B. Dalby, Tu. 1.30 |
| St. Mary's Hospital, Cambridge Place, Paddington, W. | *In- and Out-Surgeon*, Mr. G. P. Field, W. S. 9.30 |
| St. Thomas's Hospital, Westminster Bridge Road, S.E. | *Out-Surgeon*, Mr. Clutton, M. 1.30 |
| University College Hospital, Gower Street, W.C. | *In- and Out-Surgeon*, Mr. Barker, Th. 9 |
| Westminster Hospital, Broad Sanctuary, S.W. | *In- and Out-Surgeon*, Mr. Barrow, Tu. F. 9 |

### EYE DISEASES.

| | |
|---|---|
| Central London Ophthalmic Hosp., 238a, Gray's Inn Rd., W.C. Sec., Mr. G. H. Leah. Admission free | *Surgeons*, Messrs. T. Archer, G. Abbott, W. Charnley, W. Jessop, E. Clarke, J. R. Kemp and Granville Barker (House Surgeon). Daily, 1 |
| Evelina Hosp. for Sick Children, Southwark Bridge Rd. | *Surgeon*, Dr. W. Brailey, Th. 1 |
| Great Northern Central Hospital, Caledonian Rd., N. | *Surgeon*, Mr. Hutchinson, Jnr., Tu. F. 9.30 |
| Guy's Hospital, St. Thomas's Street, S.E. | *Surgeons*, Mr. Higgens, Dr. Brailey, Tu. F. 12.30 |
| Hospital for Sick Children, 49, Gt. Ormond Street, W.C. | *Surgeon*, Mr. R. Marcus Gunn, Tu. 2 |
| King's College Hospital, Portugal Street, W.C. | *Surgeon*, Mr. McHardy, M. W. Th. 1.30 |
| London Hospital, Whitechapel Road, E. | *Surgeons*, Messrs. Waren Tay, S. 9; Eve, Tu. 9 |
| Middlesex Hospital, Berners Street, W. | *Surgeon*, Mr. Lang, Tu. F. 9 |
| National Hosp. for the Paralysed, etc. Queen Sq., W.C. | *Surgeons*, Messrs. R. Brudenell Carter, F. 4; R. Marcus Gunn, Tu. 4 |
| North-West London Hosp. 18, Kentish Town Rd., N.W. | *Surgeon*, Dr. W. Collins, F. 3.30 |
| Paddington Green Children's Hosp., Paddington Grn. | *Surgeon*, Mr. W. H. H. Jessop, Th. 9 |
| Royal Free Hospital, Gray's Inn Road, W.C. | *Surgeon*, Mr. Mackinlay, M. F. 9 |
| Royal London Ophthalmic Hospital, Blomfield Street, Moorfields, E.C. Sec., Mr. R. J. Newstead. Admission free to the poor. | *Surgeons*, Messrs. J. W. Hulke, W. S.; G. Lawson, Tu. F.; J. Couper, Tu. F.; W. Tay, M. Th.; J. Tweedy, Tu. F.; E. Nettleship, W. S.; R. M. Gunn, W. S.; Lang, M. Th. Hours, 8 to 10 |
| Royal South London Ophthalmic Hosp., 6, St. George's Circus, S.E. Sec., Mr. C. Comyn. Free to the necessitous poor; or by payment | *Surgeons*, Messrs. Watson, McHardy. Daily, 2 |
| Royal Westminster Ophthalmic Hospital, King William Street, W.C. Sec., Mr. T. Beattie-Campbell. Poor and accidents free | *In- and Out-Surgeons*, Messrs. Power, M. F.; Frost, M. Th.; Rouse, Tu. S.; Hartridge, Tu. F.; Macnamara, M. Th.; Cowell, W. S.; Juler, W. S. Hour, 1 |
| St. Bartholomew's Hospital, Smithfield, E.C. | *Surgeons*, Messrs. Power, Th. 2; Vernon, Tu. S. 2.30 |
| St. George's Hospital, Hyde Park Corner, S.W. | *Surgeons*, Messrs. B. Carter, Frost, M. Th. 2; F. 1.15; W. S. 2 |
| St. Mary's Hospital, Cambridge Place, Paddington, W. | *Surgeons*, Messrs. A. Critchett, H. Juler, Tu. F. 9.30 |
| St. Thomas's Hospital, Westminster Bridge Road, S.E. | *Surgeons*, Messrs. Nettleship, Tu. Th. F. 1.30; Lawford, M. W. 1.30 |
| University College Hospital, Gower Street, W.C. | *Surgeon*, Mr. Tweedy, M. Tu. Th. F. 2 |
| West London Hospital, Hammersmith Road, W. | *Surgeons*, Messrs. B. Vernon, H. Dunn Tu. Th. S. 2 |
| Westminster Hospital, Broad Sanctuary, S.W. | *Surgeon*, Mr. Cowell, M. Th. 2 |
| Western Ophthalmic Hosp. 153 and 155, Marylebone Rd., W. Sec., Mr. G. E. Manton. By letter or small payments; free to poor | *Surgeons*, Drs. Miller, M.; Collins, F., Wheatly, M. Th.; Messrs. Carmalt Jones, W.; Buxton, Tu. F.; Nunn, W. S. Hour, 2 |

